# Home-Based Immersive Virtual Reality to Improve Motor Performance in Children and Adolescents With Developmental Coordination Disorder: Crossover Study

**DOI:** 10.2196/84995

**Published:** 2026-03-10

**Authors:** Mohammed Alharbi, David Harris, Helen Dodd, Greg Wood, Gavin Buckingham

**Affiliations:** 1 Department of Public Health and Sport Sciences, University of Exeter Medical School, University of Exeter, St. Luke's Campus, Heavitree Road, Exeter, EX1 2LU, United Kingdom, 44 1392 661000; 2 Department of Physical Therapy, Faculty of Applied Medical Sciences, University of Hail, Hail, Saudi Arabia; 3 Department of Sport and Exercise Sciences, Faculty of Science and Engineering, Manchester Metropolitan University, Manchester, United Kingdom

**Keywords:** developmental coordination disorder, DCD, game, *Movement Assessment Battery for Children, Third Edition*, MABC-3, pediatric, virtual reality, VR, extended reality, XR

## Abstract

**Background:**

Children with developmental coordination disorder (DCD) experience motor difficulties that limit daily activities and reduce physical activity enjoyment. Immersive virtual reality (VR) offers the potential for feedback-rich movement practice, but evidence for these effects in DCD remains limited.

**Objective:**

This study aims to investigate the effects of an immersive VR rhythm game compared to tablet-based gameplay within a home-based setting on motor performance, enjoyment, and motivation in children and adolescents with DCD.

**Methods:**

This crossover study included 27 participants (21 boys and 6 girls) aged 10 to 16 years with DCD who completed 2 home-based interventions, each delivered over 5 consecutive days: VR gameplay using Beat Saber (Beat Games) and tablet-based gameplay using Cut the Rope (ZeptoLab). Participants were recruited in England using convenience sampling via social media; eligible participants were aged 10 to 16 years, met research criteria for DCD based on a Developmental Coordination Disorder Questionnaire screening, and had no alternative neurological or musculoskeletal diagnosis. Each condition required at least 30 minutes of daily gameplay and was separated by a 2-week or more washout period. Motor performance was assessed pre- and postintervention using the *Movement Assessment Battery for Children, Third Edition* (MABC-3) and the Box and Block Test. Enjoyment was measured pre- and postintervention using the Physical Activity Enjoyment Scale. Participants also rated their motivation and feelings during each gameplay session. Repeated-measures ANOVAs and paired-samples 2-tailed *t* tests (α=.05) were used to examine the data.

**Results:**

For MABC-3 domains, condition × time interactions were nonsignificant, although exploratory within-condition analyses showed pre-post improvements in the VR condition. For the Box and Block Test, condition × time interactions were significant for both hands, with a greater degree of pre-post improvement in VR than tablet-based gameplay. In the VR condition, mean block transfer increased for the dominant hand (ΔM 5.93, 95% CI 3.49‐8.36; *t*_26_=−4.99; *P*<.001; Cohen *d*=0.96) and the nondominant hand (ΔM 5.11, 95% CI 2.65‐7.57; *t*_26_=−4.27; *P*<.001; Cohen *d*=0.82), whereas no significant changes were observed in the tablet condition (all *P*≥.36). VR gameplay also yielded higher enjoyment, and children reported higher motivation and feeling ratings across VR sessions than tablet sessions.

**Conclusions:**

This study, in contrast to most of the existing literature on nonimmersive technologies, examined the effect of a home-based *immersive* VR rhythm game intervention for children and adolescents with DCD. This study provides early evidence that this VR rhythm game was engaging and may support greater short-term improvements in motor performance than tablet gameplay. These findings suggest home-based immersive VR rhythm games could be a practical adjunct to pediatric rehabilitation to increase movement practice and motivation, although larger and longer trials are needed to confirm clinical impact and identify which game features drive this benefit.

## Introduction

### Background

Developmental coordination disorder (DCD), or dyspraxia, is a neurodevelopmental condition characterized by marked impairment in the acquisition and execution of coordinated motor skills that interferes with daily living and academic achievement [1,2]. Motor coordination describes a range of fundamental movement skills such as object manipulation (eg, grasping, kicking, and throwing) and locomotor activities (eg, walking, running, and jumping) [1,2]. It is an essential component of human development, supporting everyday self-care, communication, and active engagement in learning environments [1,2]. These motor skills support physical play and enable independent completion of activities of daily living [2]. For school-aged children, motor tasks represent a substantial portion of everyday academic activities [3]. Approximately 46% of children’s time spent at school involves participation in tasks requiring motor coordination, such as handwriting, coloring, cutting with scissors, and using tools [3]. Adequate motor coordination supports physical and cognitive development, psychosocial health, and overall well-being [4]. Deficits in motor skills could restrict participation, reduce quality of life, impair confidence, and adversely affect developmental outcomes [1].

Difficulties in motor coordination often appear in early childhood, typically around preschool age [5]. These difficulties become more evident in primary school as motor demands increase in daily routines [6]. DCD affects about 5% to 6% of school-aged children who often struggle with everyday tasks needed for home, school, and play [2,7]. Common difficulties include handwriting, dressing, tying shoelaces, brushing teeth, and taking part in sports and leisure activities [8]. As a result, participation is often less frequent, and children with DCD engage in a narrower range of activities than their typically developing peers [9]. These constraints affect current motor performance and broader psychological, emotional, and social development [1]. Early identification and effective intervention are therefore needed [10].

In recent years, growing research interest has emerged regarding innovative and engaging approaches aimed at enhancing motor performance and physical activities among children [11]. Immersive Virtual Reality (VR), characterized by an interactive computer-generated environment typically explored through full-body movements, has rapidly gained prominence within the consumer entertainment sector [12]. In this paper, we use the term immersive virtual reality (VR) to refer to computer-generated, 3D environments that surround the user and are typically accessed via head-mounted displays or comparable 3D displays, together with motion- or spatial-tracking technologies (eg, handheld controllers, optical tracking of hand and body parts, or other sensor-based interfaces) that enable active interaction with virtual objects and spaces [12]. In rehabilitation, VR offers a motivating, realistic setting with immediate and often intrinsic feedback [13,14], potentially increasing adherence to treatment programs [15] and facilitating the repetition of targeted motor tasks [16,17]. For children, motivation and enjoyment are key to sustaining activity [18]. VR-based games could stimulate intrinsic motivation and enjoyment, making rehabilitation feel playful and rewarding, encouraging persistent practice [19]. Studies report benefits of VR for skill acquisition in typically developing children [20] and in children with acquired brain injury [21], autism spectrum disorder [22], and cerebral palsy [17], but evidence on immersive VR for children and adolescents with DCD is scarce. A recent scoping review by Alharbi et al [23] found no studies using fully immersive VR systems in this population, indicating a clear research gap.

Exercise-based VR rhythm games, such as Beat Saber, involve physically interactive gameplay synchronized with rhythmic auditory and visual stimuli [24]. Beat Saber has a growing evidence base in motor control research, demonstrating improvements in reaction time and hand-eye coordination in typically developing youth populations [25-27]. In a recent Patient and Public Involvement (PPI) activity, we conducted a focus group where children and adolescents with DCD experienced various VR sports and rhythm games, including Beat Saber. Contributors in this activity identified Beat Saber as especially engaging because of its music movement coupling and simple controls. This rhythm game requires bimanual, goal-directed arm movements, precise timing, and rapid visuomotor responses, with graded difficulty that enables high-dose practice of coordinated actions. These features suggest that VR-based rhythm games may benefit motor skills in children and adolescents with DCD. Accordingly, the primary aim of this study was to investigate the impact of a home-based immersive VR rhythm game on motor performance, motivation, and enjoyment in children and adolescents diagnosed with or suspected of having DCD.

### PPI Activity

This study was informed by a recent PPI activity that explored the perspectives of children and adolescents with DCD on sports participation and immersive VR games [28]. Five participants aged 13 to 16 years who self-reported as having DCD took part in this study.

The PPI activity had 2 visits. The first visit involved a focus group where contributors discussed experiences and challenges in relation to traditional sports and manual tasks and commented on promotional videos of various VR sports and rhythm-based games. In the second visit, contributors took part in a hands-on VR session, followed by brief semistructured interviews about usability, enjoyment, perceived difficulty, any adverse symptoms (eg, motion sickness), content preferences, and perceived feasibility of VR for home use and rehabilitation. Contributors played VR sports titles (eg, Rezzil Index football drills, table tennis, tennis, racket sports, volleyball, and golf) and the rhythm game Beat Saber using a Valve Index VR headset.

Contributors shared a variety of opinions about the immersive VR games. Some described the games as enjoyable and accessible; others found them less engaging because of their lack of dynamic interactions and player movement. These responses suggested that engagement with immersive VR games may vary depending on individual preference and perceived challenge. Beat Saber, a rhythm-based game included in the session, was described as especially engaging and motivating. Contributors highlighted that they liked the combination of music, movement, and straightforward controls, which made the activity physically demanding yet manageable. The clear rhythmic structure and lack of competitive pressure were described as supporting enjoyment and confidence.

Insights from this PPI activity directly informed the study design, guiding the selection of this rhythm-based game as the primary VR task and supporting a home-based protocol with graded difficulty and repeated short sessions.

## Methods

### Participants

We recruited for a fixed time period of 6 months from September 2024 to March 2025; within the available recruitment period, 30 children and adolescents, aged 10 to 16 years (23 male and 7 female participants), with confirmed or suspected DCD participated in the study. With this sample size, based on an a priori power analysis conducted in G*Power (version 3.1.9.6; Heinrich Heine University Düsseldorf), using a 2-dependent-means 2-tailed *t* test with a desired power of 0.80 (*α*=.05), we would be able to detect a medium effect size (*dz*=.53). We selected the age range of 10 to 16 years on developmental, clinical, and practical grounds. Ten years was chosen as the lower bound because by around the age of 10 years, most children are in middle childhood, with sufficiently developed concrete reasoning, sustained attention, and independent reading skills to participate in structured, school-like tasks [29]. So, children of this age typically have sufficient cognitive, attentional, and reading abilities to follow a home-based protocol, complete self-report measures, and use an immersive VR headset safely. From a safety and feasibility perspective, we did not include children younger than 10 years because Tychsen and Foeller [30] found that those aged 4 to 10 years showed increased general discomfort, head-neck discomfort, fatigue, and visually induced motion sickness on the child-friendly Simulator Sickness Questionnaire measures after VR exposure compared with their pre-VR ratings. An age of 16 years was chosen as the upper bound to capture mid- to late-adolescence while still focusing on school-aged young people who are typically managed within pediatric and educational services for DCD.

Participants were recruited from England, United Kingdom, using convenience sampling via social media platforms including X (formerly known as Twitter), Facebook, and Instagram. Because recruitment occurred via open social-media platforms, the number of individuals reaching or viewing the advert is unknown, and a response rate cannot be calculated. Eligibility screening was performed using the Developmental Coordination Disorder Questionnaire (DCDQ) [31], which was completed by participants’ parents or guardians. The DCDQ is a validated screening instrument designed to assess children’s motor coordination capabilities in those aged approximately 5 to 15 years [32]. Inclusion required DCDQ scores of 57 or less on the 15-item DCDQ (total 15‐75; lower scores indicate greater difficulty); 58 to 75 is interpreted as not indicative of DCD. For adolescents aged 16 years, DCDQ scores were used as supportive screening information alongside baseline motor assessment rather than as a diagnostic indicator to determine participation in our study, and we recognize that using the DCDQ slightly beyond its validated age range may reduce screening precision for the oldest participants. Exclusion criteria were children with co-occurring neurological or musculoskeletal conditions affecting motor or cognitive function (eg, muscular dystrophy and cerebral palsy), or children who had undergone recent surgery or suffered a severe physical injury. Participants who already owned a home VR headset were excluded to reduce variability due to differences in prior exposure, unequal device familiarity, and uncontrolled use outside the prescribed days (during 2 wk or washout period of the tablet). Families meeting the inclusion criteria were invited to the research laboratory for participation.

Three participants withdrew during the interventions (2 male and 1 female participants): one during the VR condition and two during the tablet condition. Reasons for withdrawal included difficulties managing study participation alongside school commitments, disinterest (in tablet-based activities), or a lack of motivation to continue participation. Analyses were conducted on participants who completed both conditions (n=27; mean 12.7, SD 2.07; 21 male and 6 female participants). Data from withdrawals were excluded. Among these 27 participants, the baseline of the *Movement Assessment Battery for Children, Third Edition* (MABC-3) data indicated that 25 scored at ≤16th percentile on the total motor score and, together with DCDQ ≤57 and the exclusion of alternative neurological or musculoskeletal conditions, were classified as meeting research criteria consistent with the *Diagnostic and Statistical Manual of Mental Disorders, Fifth Edition* (DSM-5) for DCD. The remaining 2 participants had the MABC-3 scores in the 16th to 37th percentile range, but DCDQ<57 scores and functional motor difficulties and are therefore considered as having probable DCD. A 16th percentile or less was used as the cutoff for the total motor score in line with international clinical practice recommendations for DCD when using the MABC-3 (or equivalent objective motor measures) [33]. For brevity, we refer to this combined group as “children and adolescents with DCD” throughout the paper.

### Ethical Considerations

Ethical approval was obtained from the local research ethics board at the University of Exeter (reference: 5759511). Written informed consent was obtained from the participants’ parents or guardians, and all participating children provided their assent prior to the initiation of the study procedures. The consent or assent process made clear that the participation was voluntary and that families could withdraw at any time without giving a reason. To protect privacy and confidentiality, each participant was assigned a unique study ID, and all questionnaires and assessments were stored in deidentified form. Identifiable information (eg, names and contact details) was stored separately from research data on secure, password-protected university servers with access restricted to authorized members of the research team. Only anonymized data are reported herein. Participants received financial compensation of £50 (US $67.66) as a fixed reimbursement for their time and any travel costs. No images in the study or supplementary materials include identifiable participants.

### Study Design and Experimental Procedure

**Figure 1.**
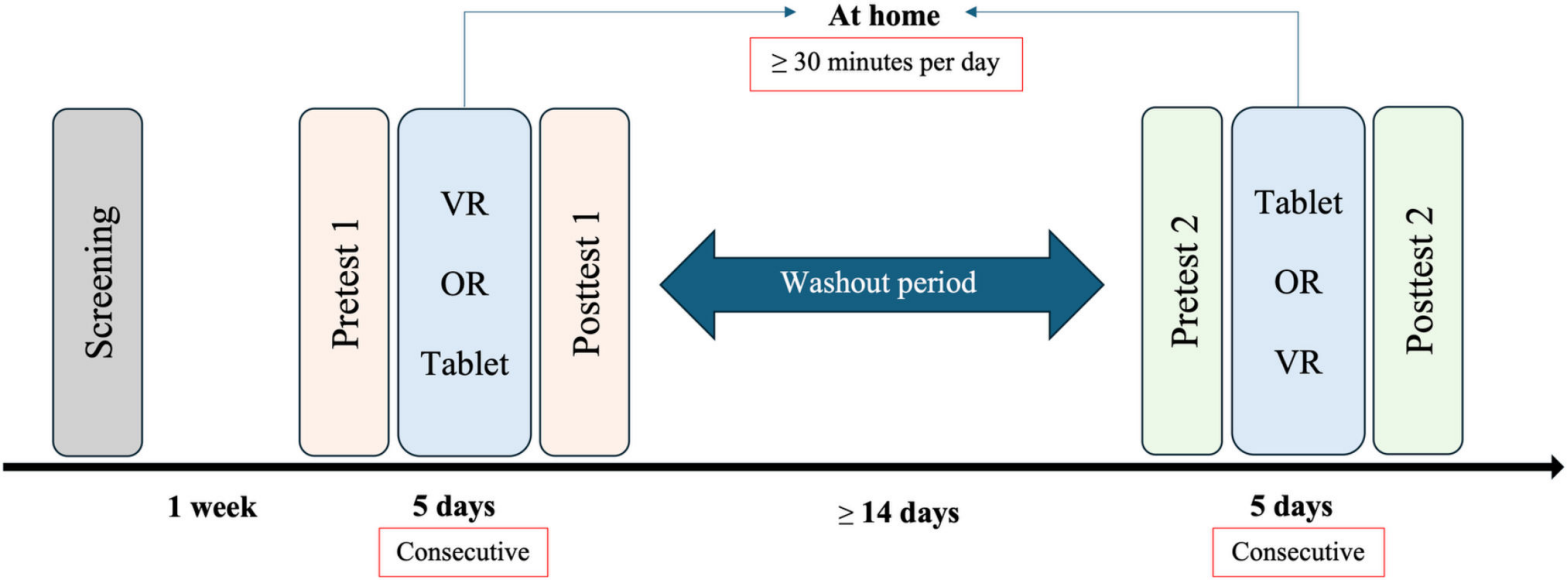
Study protocol. VR: virtual reality.

This study used a crossover design. Each participant completed two 1-week interventions: a tablet-based game (tablet condition) and Beat Saber on a VR headset (VR condition). A 5-day intervention period was chosen primarily for comparability with previous immersive VR studies using Beat Saber, which has demonstrated meaningful changes over a similar short, intensive timeframe. In particular, Grosprêtre et al [26] and Rutkowski et al [25] used Beat Saber–based protocols of less than 1 week and reported measurable improvements, suggesting that this duration is sufficient to elicit short-term effects while remaining acceptable and feasible for children and families. The order of conditions was counterbalanced by simple alternation based on enrollment: the first enrolled participant was assigned to the tablet condition first, the second to VR first, and so on. The interventions were separated by a washout period of at least 14 days. Participants were instructed to play a minimum of 30 minutes per day for 5 consecutive days at home (Figure 1). A minimum of 30 minutes per day for 5 consecutive days was chosen to provide a dose that clearly exceeds the minimum effective exposure reported in previous Beat Saber VR studies [25,26], where participants played for around 15 minutes per day over 5 days and still showed improvements. By doubling the daily playtime, we aimed to increase the volume of motor practice and the likelihood of detecting short-term effects. Adherence was monitored with a daily log completed after each session; entries recorded session completion and approximate duration. No device logs were used, so adherence reflects self-report.

Assessments were conducted both before (pre) and after (post) each intervention using the MABC-3, the Box and Block Test (BBT), and the Physical Activity Enjoyment Scale (PACES). After each gameplay session, participants were asked to report their motivation and feeling ratings in the daily log. After the postassessment, the researcher collected the device, erased participant data, and reset the game application for the next participant. Blinding was not possible because participants and caregivers were aware of the intervention being used (VR vs tablet); outcome assessments were conducted by the same assessor who was not blinded to the condition. All outcome assessments were administered using standardized instructions by a trained assessor, using the same equipment and testing environment at each visit to reduce measurement variability. This study is reported in accordance with the APA JARS-Quant (Journal Article Reporting Standards for Quantitative Research) guidelines (Checklist 1) [34].

### Interventions

#### VR Gameplay Condition

The VR condition used Meta Quest 2 headsets (Meta Platforms, Inc; 1832×1920 pixels per eye; 120 Hz) with 2 handheld controllers. Participants played Beat Saber (version 1.40.7_7060; Beat Games), a rhythm-based VR game in which colored cubes approach in sync with music. Players use 2 virtual sabers, one per hand, to slice cubes in the specified direction with the correct hand and in time to the beat, and must avoid obstacles. Scoring is based on the number of accurately sliced cubes, the precision of the slicing motion, and alignment with the rhythm of the soundtrack. Participants were instructed to begin playing the game on the default (“normal”) difficulty setting. They were further informed that if the “normal” difficulty was too challenging, they could switch to the “easy” mode. Conversely, participants who found the “normal” mode insufficiently challenging were permitted to progress to the “hard” mode. The difficulty level was not systematically tracked during the intervention. This game was considered to be a promising intervention due to its emphasis on whole-body motor coordination, bilateral upper-limb control, visuomotor integration, and rhythmic timing [25,26], skills that are commonly affected in children with DCD [7]. Prior studies have highlighted this game as an engaging platform for motor training, offering real-time feedback and adaptive difficulty levels [35,36].

Participants completed the VR gameplay at home under caregiver supervision, in accordance with the safety instructions provided. Each caregiver received a detailed guidance sheet outlining safety protocols, including monitoring for motion sickness, ensuring a clear play area, and being present during all gameplay sessions (VR instructions can be located online) [37]. Prior to the VR intervention, each participant received a brief orientation on how to use the VR headset and interact with the game. Adherence was monitored through self-report logs, in which participants or caregivers recorded daily gameplay duration and any issues encountered. Any adverse effects, such as motion sickness or fatigue, were also noted. No such incidents were reported.

#### Tablet Gameplay Condition

The tablet condition used a Lenovo Tab M8 (fourth generation) Android tablet with an 8-inch LCD (1280×800) display and dimensions of 197.97×119.82×8.95 mm. Participants played Cut the Rope (version 3.75.0; Zeptolab), a puzzle game that requires cutting virtual ropes in a planned sequence to deliver candy to the character Om Nom. The game was administered using the default settings. In this mode, levels are presented sequentially, with successful completion of a level automatically unlocking the next one. Puzzle complexity increases gradually (eg, greater numbers of ropes, obstacles, and timing constraints), but there is no separate user-adjustable difficulty setting or fixed level cap relevant to the amount of play time in this study. Players can repeat the same level as many times as needed until it is solved. Therefore, if they become stuck, progression may slow, and they may remain on earlier levels for longer. We selected this game as an active control that emphasizes problem-solving with minimal motor demands and has been used as a control in DCD research [38]. The game is self-paced and does not include rhythm- or music-based timing mechanics, and actions are not performed in synchrony with a beat or external tempo.

As with the VR condition, gameplay for the tablet condition occurred at home, and participants were instructed to play for a minimum of 30 minutes daily across 5 consecutive days. Prior to the tablet intervention, each participant received a brief orientation on how to navigate and interact with the game. Caregivers were not asked to supervise sessions. Participants were given the tablet directly and expected to engage with the game independently. Engagement and adherence were recorded in daily logs. The application was reset after each participant to protect data privacy.

In both conditions, children and parents were asked to “Play at least 30 minutes per day,” but were explicitly told there were no “right” or “wrong” amounts of play, that missing or shortening sessions were acceptable if they were tired, busy, or unwell, and that honest recording was more important than appearing highly adherent. They were also reminded that logs were for research only, anonymized, and not shared with anyone else.

### Outcome Measurements

#### Movement Assessment (MABC-3)

The primary outcome was performance on the MABC-3, a standardized, norm-referenced assessment of motor coordination for ages 3 to 25 years [39]. The MABC is widely used in clinical and research settings to identify motor difficulties and monitor development [40]. The MABC-3 includes 10 subtests across 3 domains: manual dexterity, aiming and catching, and balance and locomotion. Tasks were administered by age band per the manual (7‐11 y=band 2; 12‐25 y=band 3). Trained assessors followed the standardized protocol. Testing took place in a quiet laboratory room using standard MABC-3 equipment as specified in the manual. Raw scores from each item were converted to standard scores using age-referenced normative data. A total motor score was computed by summing the standard scores from each domain, providing an overall estimate of motor proficiency. The MABC-3 scores are interpreted using percentile ranks. Consistent with published guidelines, scores above the 16th percentile are considered indicative of typical motor performance; scores between the 6th and 15th percentile suggest a risk of motor coordination difficulties, and scores at or below the 5th percentile are classified as indicative of significant motor coordination impairment. Although MABC-3 is a newer version of the widely validated *Movement Assessment Battery for Children, Second Edition* (MABC-2), both editions have demonstrated strong psychometric properties, including high test–retest reliability and construct validity for use in pediatric populations [39]. Its application in this study was chosen due to its relevance for identifying motor coordination challenges commonly observed in children with DCD.

#### Upper-Limb Manual Dexterity Assessment (BBT)

The secondary outcome was performance on the BBT, a standardized measure of unilateral gross manual dexterity [41]. The apparatus is a wooden box with 2 equal compartments (53.7×25.4×8.5 cm), a central partition, and 150 wooden blocks with sides measuring 2.5  cm [42]. Participants are asked to transfer as many blocks as possible, one at a time, from one compartment to the other within 60 seconds using a single hand.

Administration followed the standardized guidelines described by Mathiowetz et al [42]. Each hand received a 15-second practice trial, followed by one 60-second timed trial. Testing was completed for the dominant hand first, then the nondominant hand, at each pre- and postsession. Hand dominance was determined by self-report. Scores were the number of blocks transferred per hand; if multiple blocks were moved at once, only one was counted. The BBT shows high test-retest reliability in pediatric samples [43,44]. The BBT was administered before and after each intervention (VR and tablet), and dominant and nondominant hand scores were analyzed separately. The BBT was selected as a primary measure of unimanual gross manual dexterity because fine and gross upper-limb coordination are commonly impaired in children with DCD. The BBT was selected as a measure of unimanual gross manual dexterity because fine and gross upper-limb coordination are commonly impaired in children with DCD and are central to our motor performance objectives. This test has been used in other studies quantifying motor performance in this population [45].

#### Enjoyment of Physical Activity (PACES)

PACES was used to assess enjoyment during each intervention [46]. A child-appropriate 16-item version validated for pediatric physical activity research was administered [47-49]. At baseline, PACES was administered with a generic physical-activity stem, with items phrased as “when I am doing physical activity...” (eg, “I enjoy it” and “It’s very exciting”). After each intervention block, PACES was then readministered with condition-specific wording (“when I am playing the VR game...” / “when I am playing the tablet game...”). The questionnaire included both positively and negatively worded items (eg, “It makes me feel depressed” and “It gives me energy”), with reverse scoring applied to negatively worded items before analysis. Children were instructed to reflect on their experience during the specific condition and respond accordingly. Higher scores indicated greater enjoyment. The questionnaire was administered electronically via Qualtrics (Qualtrics, LLC) [50]. A template of the PACES questionnaire can be located online [51].

The PACES questionnaire was completed before (pre) and after (post) each condition (VR and tablet) to account for baseline expectations and effects that can distort post scores. While enjoyment is commonly assessed postintervention, relying solely on postintervention scores can obscure the effect of the activity itself, particularly if participants hold strong initial expectations or attitudes. This approach was informed by methodological concerns raised in related domains, such as tolerability studies using the Simulator Sickness Questionnaire, where assuming a symptom-free baseline has led to inaccurate conclusions [52]. It is worth noting that children may have varying levels of enjoyment or anticipation before gameplay, meaning that not all postintervention responses can be attributed solely to the gameplay experience. Capturing enjoyment both before and after each condition allowed us to account for individual baseline affect and more accurately assess the impact of the intervention. If a child requested help, the assessor or a parent read items aloud and clarified word meanings using neutral definitions, and the child selected their own response.

#### Motivation and Feeling

Daily motivation and feeling during gameplay were recorded using a child-friendly log adapted from Scott et al [38] for home-based DCD interventions. The adapted “Motivation and Feeling Log” (a template can be located online) [53] was aligned to the 2 conditions (VR and tablet). Each participant completed a 5-day log for each condition immediately after each session. Motivation was rated on a 5-point Likert scale (1=“very low” to 5=“very high”), with an option to add brief comments. Feeling was captured by choosing one of a series of emoji-style faces (very unhappy to very happy) and by responding to short open-ended prompts (eg, “Tell us how you feel it went” and “Did you enjoy the tasks?”).

### Statistical Analyses

All analyses were conducted in Jamovi (version 2.6.44; The Jamovi project) and R (version 4.2.2; R Foundation for Statistical Computing; R.app v. 1.79, macOS Big Sur ARM). The per-protocol sample (n=27) was used for all inferential tests. There were no missing outcome data among participants who completed both conditions; therefore, no imputation was performed. For MABC-3 and BBT outcomes, 2×2 within-subjects ANOVAs tested effects of time (pre and post) and condition (VR, tablet). In addition to the primary ANOVAs, exploratory within-condition paired-samples 2-tailed *t* tests were conducted to examine pre-post changes in each outcome. Exploratory within-condition tests were not adjusted for multiple comparisons and are interpreted cautiously. For motivation and feeling across days, a 2×5 repeated-measures ANOVA tested condition (VR and tablet) by day (1-5). Assumptions of normality (Shapiro-Wilk) and homogeneity of variance (Levene test) were tested in Jamovi. Effect sizes were reported as Cohen *d* for paired comparisons and partial *η²* for ANOVA terms. Statistical significance was set at *P*<.05 for all analyses.

## Results

**Figure 2.**
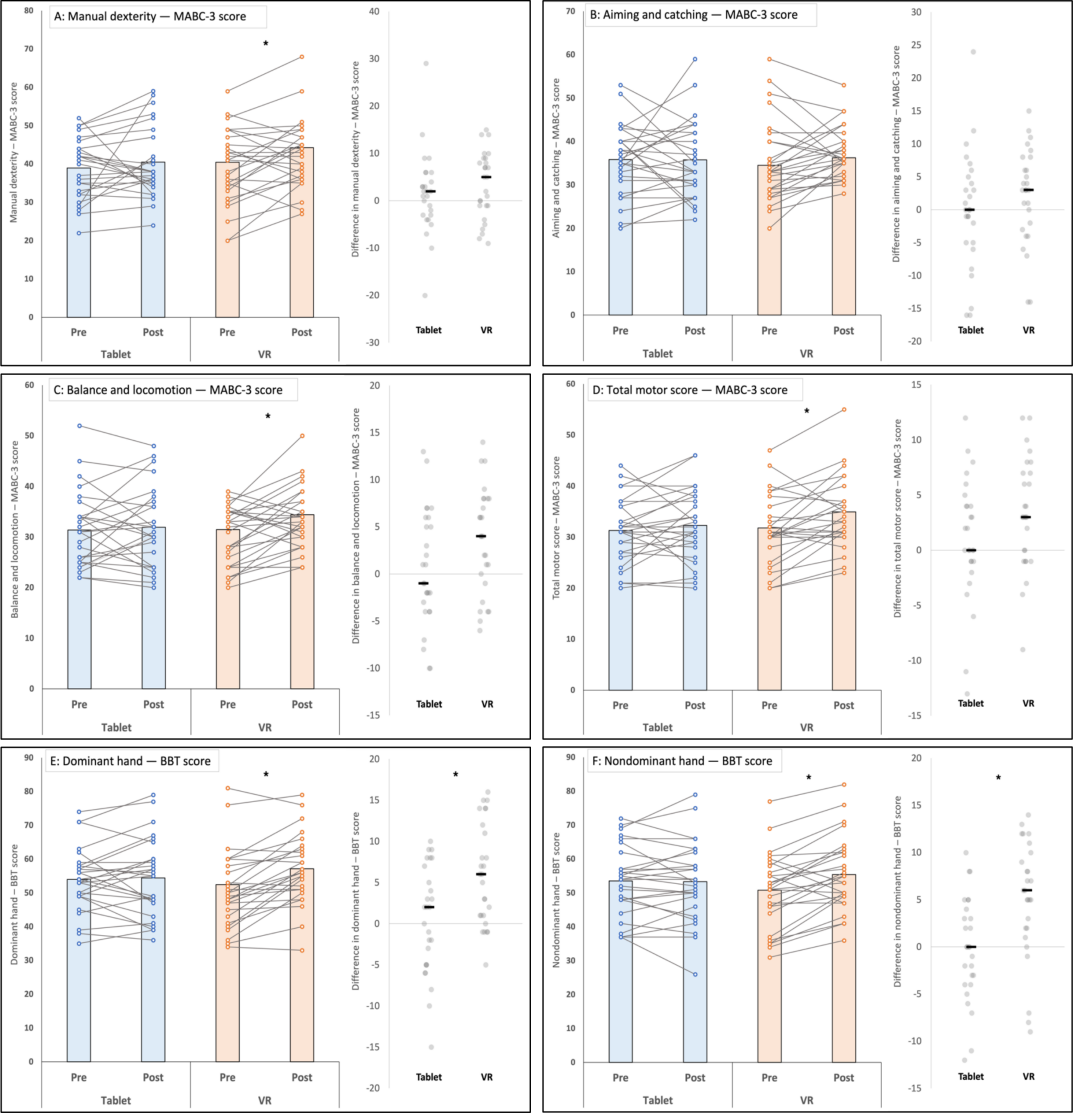
Within-participant pre-post motor performance across tablet and virtual reality (VR) conditions: (A) manual dexterity*—Movement Assessment Battery for Children, Third Edition* (MABC-3), (B) aiming and catching—MABC-3, (C) balance and locomotion—MABC-3, (D) Total Motor Score—MABC-3, (E) dominant hand—Box and Block Test (BBT), and (F) nondominant hand—BBT. Asterisks indicate statistically significant differences (*P*<.05).

A total of 30 participants enrolled in this study; 3 withdrew during the intervention period (one during VR and two during tablet), leaving 27 participants who completed both conditions and were included in the per-protocol analyses. Repeated-measures ANOVAs examined condition (VR, tablet)× time (pre and post) effects on motor performance outcomes. For brevity, here we report on the interactions—the reporting of the main effects can be found in a study by Alharbi et al [54]. Interactions were nonsignificant for MABC-3 domains: manual dexterity (*F*_1,25_=0.81; *P*=.38; *η²_p_*=0.03; Figure 2A), aiming and catching (*F*_1,25_=0.83; *P*=.37; *η²_p_*=0.03; Figure 2B), balance and locomotion (*F*_1,25_=2.42; *P*=.13; *η²_p_*=0.09; Figure 2C), and total motor score (*F*_1,25_=3.36; *P*=.08; *η²_p_*=0.19; Figure 2D). Significant interactions were observed for the BBT dominant hand (*F*_1,25_=7.47; *P*=.01; *η²_p_*=0.23; Figure 2E) and BBT nondominant hand (*F*_1,25_=11.94; *P*=.002; *η²_p_*=0.32; Figure 2F). Estimated marginal means (95% CIs) for each outcome by condition (tablet vs VR) and time point (pre vs post) are summarized in Table 1.

**Table 1.** Estimated marginal means (95% CIs) for outcomes by condition (tablet vs virtual reality [VR]) and time point (pre vs post).

Outcome	Tablet		VR	
	Pre, mean (95% CI)	Post, mean (95% CI)	Pre, mean (95% CI)	Post, mean (95% CI)
MABC-3^a^				
Manual dexterity	39.0 (35.8‐42.2)	40.5 (36.8‐44.2)	39.4 (35.5‐43.2)	42.6 (39.1‐46.2)
Aiming and catching	35.8 (32.6‐39.1)	35.7 (32.2‐39.2)	35.2 (31.4‐39.1)	37.3 (35.0‐39.7)
Balance and locomotion	31.3 (28.4‐34.2)	31.9 (28.8‐35.0)	30.2 (27.9‐32.6)	33.7 (31.3‐36.2)
Total motor score	31.3 (28.7‐33.9)	32.3 (29.5‐35.1)	31.5 (28.8‐34.2)	34.8 (32.0‐37.5)
BBT^b^				
Dominant hand	54.0 (50.2‐57.8)	54.4 (49.8‐59.1)	50.8 (46.5‐55.1)	56.6 (52.5‐60.8)
Nondominant hand	53.5 (49.3‐57.8)	53.3 (48.6‐58.0)	50.5 (46.1‐55.0)	55.7 (51.6‐59.9)
PACES^c^				
Enjoyment	62.8 (59.5‐66.0)	53.6 (48.3‐58.9)	62.1 (58.9‐65.3)	63.2 (59.1‐67.4)

a MABC-3: *Movement Assessment Battery for Children, Third Edition.*

b BBT: Box and Block Test.

c PACES: Physical Activity Enjoyment Scale.

Although no significant interactions were observed in the ANOVA with MABC-3 domains as the dependent variable, exploratory post hoc within-condition (paired-samples) *t* tests were conducted to explicitly examine performance improvements in the VR and tablet conditions. Paired-samples 2-tailed *t* tests revealed significant pre-post improvements in the VR condition across the MABC-3 domains of manual dexterity (ΔM 3.3, 95% CI 0.51‐6.08; *t*_26_=−2.43; *P*=.02; Cohen *d*=0.47; Figure 2A); balance and locomotion (ΔM 3.52, 95% CI 1.3‐5.74; *t*_26_=−3.25; *P*=.003; Cohen *d*=0.63; Figure 2C), and total motor score (ΔM 3.33, 95% CI 1.34‐5.32; *t*_26_=−3.44; *P*=.002; Cohen *d*=0.66; Figure 2D). No significant change was found for the aiming and catching domain (ΔM 2.15, 95% CI −0.72 to 5.01; *t*_26_=−1.54; *P*=.14; Cohen *d*=0.30; Figure 2B). For the BBT in the VR condition, significant improvements were found for both the dominant hand (ΔM 5.93, 95% CI 3.49‐8.36; *t*_26_=−4.99; *P*<.001; Cohen *d*=0.96; Figure 2E) and the nondominant hand (ΔM 5.11, 95% CI 2.65‐7.57; *t*_26_=−4.27; *P*<.001; Cohen *d*=0.82; Figure 2F). By contrast, there were no significant pre-post changes observed across any MABC-3 domains or BBT performance in the tablet condition (all *P*≥.36).

**Figure 3.**
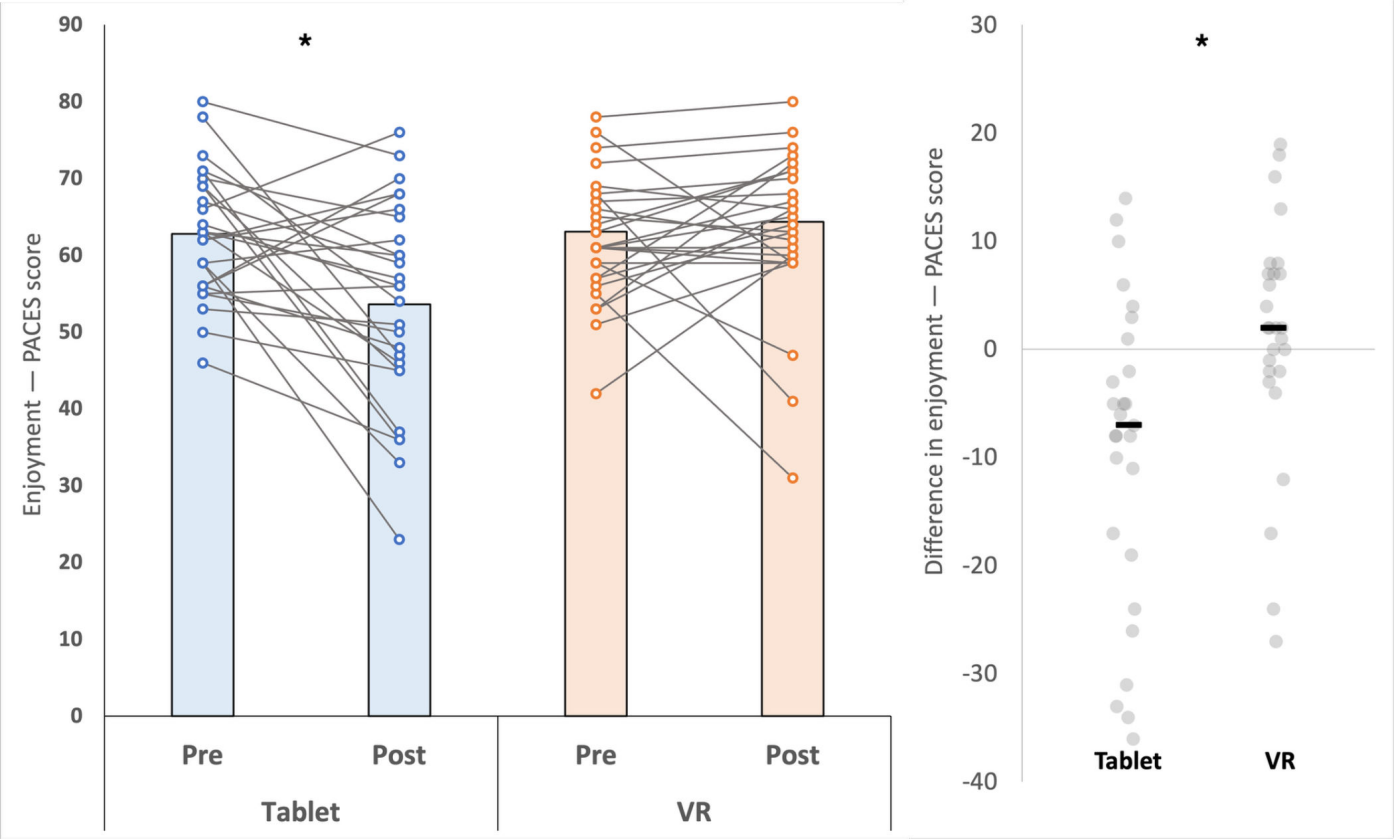
Within-participant pre-post enjoyment score (Physical Activity Enjoyment Scale [PACES]) across tablet and virtual reality (VR) conditions. Asterisks indicate statistically significant differences (*P*<.05).

For enjoyment (PACES), a condition × time interaction was detected (*F*_1,26_=11.67; *P*=.002; *η²_p_*=0.31; Figure 3). Enjoyment decreased from pre to post in the tablet condition (ΔM −9.19, 95% CI −14.79 to −3.58; *t*_26_=3.37; *P*=.002; Cohen *d*=0.65) and did not change in the VR condition (ΔM 1.11, 95% CI −3.27 to 5.49; *t*_26_=−0.52; *P*=.61; Cohen *d*=0.10).

**Figure 4.**
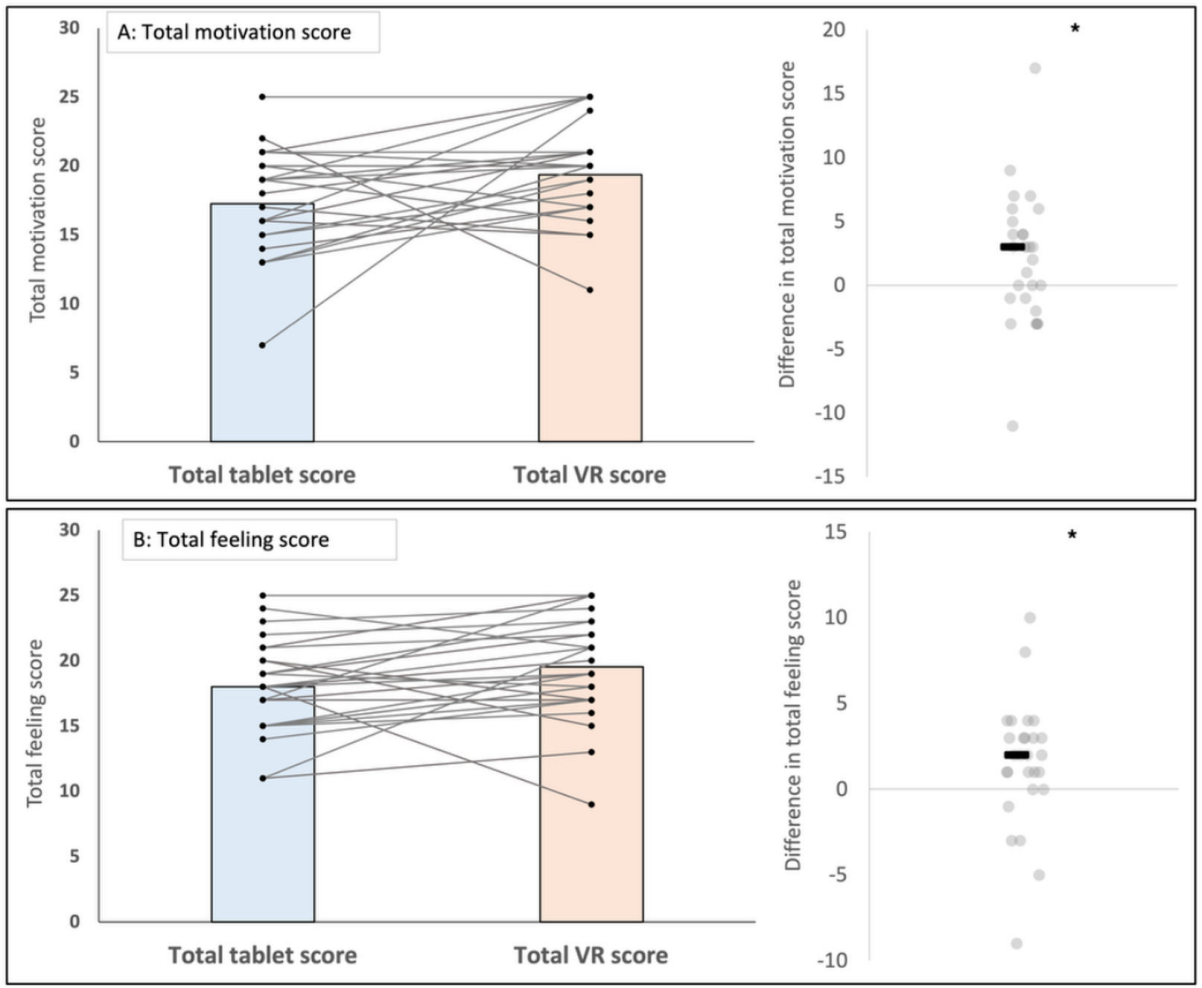
Effect of condition (virtual reality [VR] vs tablet) on self-reported outcomes: (A) motivation and (B) feeling. Asterisks indicate statistically significant differences between the VR and tablet conditions (*P*<.05).

Across the 5 days, there was no condition × day interaction for motivation (*F*_4,104_=0.54; *P*=.71; *η²_p_*=0.02), or feeling (*F*_4,104_=0.26; *P*=.91; *η²_p_*=0.01). Main effects of condition were present, suggesting that motivation was higher in VR than tablet (*F*_1,26_=4.49; *P*=.04; *η²_p_*=0.15); estimated marginal means on the 1 to 5 scale were VR 3.87 (95% CI 3.60‐4.15) versus tablet 3.45 (95% CI 3.15‐3.75). Levels of positive feeling were also higher in VR than tablet (*F*_1,26_=4.54; *P*=.04; *η²_p_*=0.15); estimated marginal means were VR 3.90 (95% CI 3.60‐4.21) versus tablet 3.60 (95% CI 3.32‐3.88; Figure 4A-B).

There was no evidence that the order of conditions, nor the duration of the washout period following the VR task, affected performance on the second session [54].

The raw data, full analysis figures, and optional session comments from the daily logs are available on Open Science Framework [55,56,57].

## Discussion

### Principal Findings

This study investigated the effects of an immersive VR rhythm game on motor performance, motivation, and enjoyment in children and adolescents with DCD. The findings highlight immersive VR rhythm games as a promising tool for movement skill development, addressing a documented gap in the literature regarding immersive VR interventions for children and adolescents with DCD [23]. Our findings tentatively suggest that immersive VR rhythm games could be associated with improvements in various aspects of motor performance and may substantially enhance intrinsic motivation and emotional engagement in children with DCD, offering a supportive approach to traditional therapeutic approaches.

The key pattern in the data provides preliminary evidence of improvement in several domains of motor performance in children and adolescents with DCD following a short-term, home-based immersive VR rhythm game. Exploratory within-condition analyses indicated that participants in the VR condition showed pre-post improvements in manual dexterity, balance and locomotion, and total motor score on the MABC-3; however, the condition × time interactions were non-significant. Consistent with this, the MABC-3 estimated marginal means showed wide and overlapping 95% CIs (Table 1), indicating limited precision for between-condition differences over this short period. Therefore, any differential effects of VR versus the tablet condition on these outcomes should be interpreted cautiously. These patterns were more clearly supported for upper-limb motor skills, where significant condition × time interactions and within-condition changes in BBT scores for both the dominant and nondominant hands suggested greater gains following VR than tablet play. The improvements in manual dexterity and bilateral upper-limb control, as evidenced by the MABC-3 and BBT results, are particularly noteworthy given that these are common areas of difficulty for children with DCD [2,7]. Taken together, these findings tentatively support the practical utility of immersive VR as a potentially effective tool for improving functional motor skills required for daily living and academic activities in children and adolescents with DCD [1,3,58].

Descriptively, the tablet condition failed to elicit any significant pre-to-post changes across any MABC-3 domains or BBT performance (all *P*>.35). This divergence in outcomes is consistent with, although does not definitively establish, the idea that the observed motor improvements are not merely attributable to general practice effects, maturation, or nonspecific engagement with a digital device. Instead, the pattern of results tentatively suggests that features of the immersive VR game may have contributed to the observed changes in motor performance. However, the present study cannot determine whether these effects are driven primarily by the immersive VR context itself, the rhythmic, music-synchronized gameplay, the whole-body movement demands, or a combination of these elements. The overall pattern is broadly consistent with prior evidence indicating that VR can support motor skill development across other pediatric conditions, including cerebral palsy and autism spectrum disorder [17,21,22]. Moreover, this study extends these benefits specifically to children and adolescents with DCD, where empirical research on immersive VR has been limited [23]. The nonsignificant condition × time interactions for the MABC-3 domains mean that the between-condition differences in pre-post change on these measures should be interpreted cautiously. Several factors may explain the lack of interaction, including the modest sample (n=27), the relatively short exposure, variable levels of motivation and fatigue of participants with assessments often conducted after school, and reliance on self-reported adherence to the VR and tablet interventions. However, the significant condition × time interactions identified for BBT scores for both the dominant hand and the nondominant hand provide evidence that, for this outcome, the immersive VR rhythm game improved participants’ motor performance more than the tablet intervention. This finding might show that Beat Saber specifically targets the rapid whole-arm coordination that underpins the BBT.

Beyond motor performance, this study also highlights the potential impact of immersive VR rhythm games, like Beat Saber, on the subjective experiences of motivation and enjoyment in children and adolescents with DCD. Participants in the VR condition consistently reported higher motivation scores and more positive emotional experiences when compared to the tablet condition. Enjoyment is closely linked to persistence, as children are more likely to continue with physical activity and learning tasks when they experience them as fun rather than purely effortful or externally pressured [18,19]. The capacity of immersive VR rhythm games to transform these experiences into intrinsically motivating activities may therefore offer a significant clinical advantage. Further emphasizing this distinction, the PACES scores revealed a significant decrease in enjoyment after the tablet condition, whereas no significant change in enjoyment was observed in the VR condition. This suggests that the tablet game’s capacity to sustain enjoyment over the intervention period was limited. Moreover, the tablet game lacked the physical interactivity, multisensory stimulation, and immersive qualities that characterize the VR experience. This absence of immersive presence and direct physical engagement likely contributed to the decline in enjoyment, highlighting a fundamental difference in the experiential quality offered by the 2 modalities. This is further illustrated by a parent’s observation:

*This is interesting... [their child] played with the tablet on the way home [two days ago] but yesterday she didn't ask for it even though it was clearly in sight on the kitchen table whereas when she had the VR she was counting down the hours when she could go on it.*

This observation supports the idea that immersive VR rhythm games led to more consistent and higher levels of engagement. These findings are also consistent with early feedback from the preliminary PPI activity, where participants expressed a preference for VR-based activities. However, a parent reported that their child used the tablet “on the way home,” indicating that, in at least some cases, the tablet was used outside the intended “home-based” context. This raises the possibility that either the home-use instructions were not sufficiently clear or that adherence varied between participants. Importantly, this context difference could have influenced both the amount and quality of engagement in a direction that may confound comparisons between conditions. For example, tablet use during travel may have occurred in shorter, fragmented sessions and/or alongside other activities (eg, conversations, distractions, and fatigue), which could reduce sustained attention and engagement quality despite increasing opportunities to play. Conversely, the greater portability of the tablet could increase total exposure time relative to VR by enabling additional ad hoc play. Either pattern could bias engagement-related outcomes and any dose–response relationship independent of the game content itself. Future studies should therefore strengthen monitoring and standardization of context (eg, clearer instructions specifying location and timing, brief daily logs of where or when sessions occurred, or objective in-app time-stamps) and, where feasible, match interventions on portability or include context-of-use as a planned covariate.

Several features of the immersive VR rhythm task plausibly underlie both the motor improvements and the higher motivation and enjoyment observed. The Beat Saber game demands precise, bilateral upper-limb coordination, rapid visuomotor integration, and accurate rhythmic timing, skills commonly affected in DCD [24,25]. High-dose, task-specific practice with immediate visual, auditory, and haptic feedback supports error detection and correction, consistent with motor-learning principles [16,17,35,36]. Moreover, the adaptive difficulty levels within the game, allowing participants to progress from “normal” to “hard” or regress to “easy” mode, align with principles of optimal challenge, ensuring that the task remains sufficiently demanding yet achievable, thereby maximizing learning [59], but we did not formally titrate or verify that the task was optimally matched to each child’s capacity. It is therefore unclear to what extent the overall difficulty was fine-tuned for individual participants with DCD. The immersive nature of VR creates a powerful sense of “being there” (presence), which can significantly enhance engagement and reduce the perception of effort associated with physical activity [12,19]. This transformation of therapeutic exercise into a playful and rewarding experience is particularly potent for children, who are naturally drawn to engaging and interactive activities [60]. The coupling of synchronized music, rhythmic movement, and immediate, clear feedback provides an intrinsically rewarding loop that fosters competence and self-efficacy [23-26,61]. Together, these properties can improve upper-limb coordination and related motor performance, and also sustain motivation and positive affect across sessions [25-27,60]. A participant’s remark illustrates this alignment:

*Felt confident on harder levels. Coordination (hand-eye) is getting better. I found the tasks [VR’s one] pleasurable and exciting, up to my growth*.

This suggests that the level progression matched their developing skill level and maintained engagement. Thus, this adaptability may have supported adherence and sustained participation, which are common barriers in traditional rehabilitation programs [19,24,27]. By contrast, although the tablet game (Cut the Rope) also includes level progression, increases in difficulty primarily reflect greater puzzle complexity rather than changes in movement demands, and the game lacks the physical interactivity, multisensory feedback, and immersive presence of VR. These differences in the type of challenge and the absence of immersive, movement-based engagement are consistent with the tablet game being experienced as lower in direct physical demand and less engaging overall. Future work could examine how in-game performance relates to improvements in movement assessment scores to shed light on the mechanisms that underpin these effects.

At the same time, participants’ comments also highlighted important drawbacks and limits to the acceptability of immersive VR. Not all children enjoyed or tolerated the VR condition as some described the headset as heavy or uncomfortable (eg, “I don’t like the game and the headset [was] heavy”), and one participant characterized the experience as “too stressful.” Another child reported that their “head just felt like it was going to explode even though Mum helped clean the VR lens and adjust the straps” and that they did not feel they had improved. Other comments referred to a loss of enthusiasm and concentration towards the end of the week, or a dislike of the repetitiveness of the game and its music, even when parents perceived some improvement. Together, these accounts indicate that immersive VR is not universally acceptable or tolerable for all children with DCD, and that issues such as physical discomfort, perceived stress, visual or cognitive overload, and content-specific preferences (eg, music and repetition) can limit engagement [62]. These quotes warrant more cautious conclusions about immersive VR and highlight the importance of flexible VR content that can accommodate differing preferences and tolerance levels [63].

### Limitations and Future Directions

Despite the promising findings, this study is not without limitations that warrant consideration for future research. First, the relatively small sample size might constrain the generalizability of the findings to the broader population of children and adolescents with DCD. The sample included more boys than girls, which is common in DCD cohorts but may also limit the generalizability of this intervention [64]. Although the within-subject crossover design mitigates the risk that this imbalance biased the comparison between VR and tablet conditions [65], the study was not powered to examine gender as a moderator, and we cannot determine whether interest, engagement, or motor outcomes differed meaningfully between boys and girls. Future work should purposively recruit more girls and formally test gender-related differences in response to immersive VR interventions. Second, the intervention duration of five consecutive days, while demonstrating acute effects, may not be sufficient to capture long-term motor learning, skill retention, or sustained behavioral changes. Some gains, particularly in motivation and enjoyment, may reflect novelty, since VR is new to most children compared with tablets. We do not know whether this motivation difference would persist once the novelty of the intervention declines [66]. Longitudinal studies with extended intervention periods and follow-up assessments are crucial to ascertain the durability of the observed motor improvements and the persistence of enhanced motivation and enjoyment. Third, the home-based nature of the intervention posed challenges for direct supervision and objective compliance monitoring. Although caregiver and self-report logs were used, these are susceptible to social desirability bias [67]. Future studies should integrate objective measures of engagement and adherence, such as in-game metrics, wearable sensor data, or remote video monitoring, to provide more accurate and verifiable data on intervention fidelity [68]. Fourth, only one immersive VR rhythm game (Beat Saber) was used, so the findings may not generalize to all immersive VR content; whether other immersive VR games without rhythm-based mechanics yield similar benefits remains to be tested. This study, therefore, cannot determine whether the observed effects are driven primarily by immersion, rhythmic or music-synchronized gameplay, whole-body movement demands, or their combination. Future research could address this by using multiple comparison conditions (eg, nonimmersive rhythm games, immersive nonrhythm VR games, or rhythm-based games with different movement demands) to help disentangle the contribution of immersion, rhythm, and whole-body engagement. Fifth, the tablet game (Cut the Rope) was intentionally chosen to minimize motor demands, but it may be more passive than the VR rhythm task. As a result, between-condition differences may simply reflect a greater ‘dose’ of movement, rather than effects specific to immersive VR. Future trials could include an active, movement-based nonimmersive control (eg, a screen-based rhythm game) matched on session length and movement intensity, verified with telemetry or accelerometry or heart-rate monitoring, to better isolate VR-specific contributions. Sixth, assessments frequently occurred after school, introducing variability in fatigue and attention. Future work, therefore, should standardize testing times or record time-of-day and brief fatigue or attention ratings so these factors can be controlled [69]. Finally, because multiple exploratory within-condition tests were conducted in MABC-3 domains after a nonsignificant interaction, the risk of type I error is increased, and these findings should be interpreted with caution [70].

### Conclusions

This study is the first to evaluate a home-based immersive VR rhythm game for children and adolescents with DCD in comparison to a tablet-based control condition with a crossover design. Prior literature in this area has focused on nonimmersive VR and provides limited direct evidence comparing immersive VR rhythm gameplay against nonimmersive gameplay with a similar practice dose. Our findings add early evidence that immersive VR rhythm gameplay can be highly engaging and may support short-term gains in manual dexterity relative to tablet gameplay. In real-world terms, these games could be a scalable home–based supplement to conventional therapy to increase practice opportunities and enjoyment; future trials should use larger samples, longer follow-up, objective usage or context tracking, and multiple comparison conditions to disentangle the effects of immersion, rhythm, and whole-body movement.

## Supplementary material

10.2196/84995 Checklist 1 JARS-Quant checklist.

## Abbreviations

BBT: Box and Block Test
DCD: developmental coordination disorder
DCDQ: Developmental Coordination Disorder Questionnaire
DSM-5: *Diagnostic and Statistical Manual of Mental Disorders, Fifth Edition*
JARS-Quant: Journal Article Reporting Standards for Quantitative Research
MABC-2: *Movement Assessment Battery for Children, Second Edition*
MABC-3: *Movement Assessment Battery for Children, Third Edition*
PACES: Physical Activity Enjoyment Scale
PPI: Patient and Public Involvement
VR: virtual reality
